# Gender Differences in Ultra-Processed Food Consumption and Its Association with Obesity Among Korean Adults

**DOI:** 10.3390/nu17122027

**Published:** 2025-06-18

**Authors:** Seung Jae Lee, Kyung Won Lee

**Affiliations:** 1 Department of Food Science and Nutrition, Yongin University, Yongin 17092, Republic of Korea; sjlee@yongin.ac.kr; 2 Department of Home Economics Education, Korea National University of Education, Cheongju 28173, Republic of Korea

**Keywords:** Korean adults, ultra-processed food, obesity, gender differences, dietary intake

## Abstract

Background/Objectives: This study aimed to examine the association between ultra-processed food (UPF) consumption and obesity in Korean adults. Methods: Data from the 2019 to 2021 Korea National Health and Nutrition Examination Survey were analyzed. Dietary intake and UPF consumption were assessed using the NOVA food classification based on 24 h recall data from 9662 participants (aged 19–64 years). The participants were divided into three groups based on the tertile of UPF intake. Obesity was defined as a body mass index of ≥25 kg/m^2^. Multivariable logistic regression was used to estimate the adjusted odds ratios (AORs) with 95% confidence intervals (CIs) after controlling for potential confounders. Results: Of the participants, 50.2% were men and 49.8% were women. Men consumed more UPFs daily (401.3 g) than women (260.1 g; *p* < 0.0001). Among the various categories of ultra-processed foods, ‘soft drinks, fruit and vegetable drinks’ were the most frequently consumed by both men and women, with men showing a notably higher intake than women. Compared to the lowest quartile of UPF intake, the highest tertile was significantly associated with obesity in men (AOR: 1.28; 95% CI: 1.05–1.55; *p* for trend = 0.0003). Conclusions: This study suggests that a high intake of UPFs is associated with increased odds of obesity in men. Further research is needed to elucidate the specific negative health effects of UPFs in different populations, and targeted efforts should promote healthy diets for both men and women.

## 1. Introduction

The term ultra-processed food (UPF) was first introduced by Monteiro with the development of the NOVA food classification system [1]. The NOVA system is a widely used framework that categorizes foods according to the extent and purpose of industrial food processing [2]. It classifies foods based on the physical, biological, and chemical processes involved throughout the entire journey of food, from its extraction from nature to its eventual consumption by individuals [3]. It classifies foods into four distinct groups according to the degree of processing: NOVA 1—unprocessed or minimally processed foods; NOVA 2—processed culinary ingredients; NOVA 3—processed foods; and NOVA 4—ultra-processed foods [2,4]. Ultra-processed foods are industrial formulations typically composed of five or more ingredients, including substances not commonly used in culinary preparations, such as flavor enhancers, colorants, emulsifiers, and other additives intended to imitate or enhance sensory qualities [5]. The consumption of processed foods is increasing globally, largely because of their convenience, extended shelf life, and enhanced palatability, which make them more accessible and appealing to consumers [6].

Increased exposure to highly processed foods has been associated with a heightened risk of adverse health outcomes, particularly cardiometabolic diseases, mental health disorders, and all-cause mortality [7]. Ultra-processed food consumption has also been linked to a higher prevalence of overweight and obesity in adults [8], increased cardiometabolic risk [9], type 2 diabetes [10,11], and hypertension [12]. Diets high in ultra-processed foods tend to promote excessive energy intake, largely because of their elevated content of refined carbohydrates and fats [13]. Consumption of refined carbohydrates and added sugars increases the risk of type 2 diabetes [14], while excessive intake of added and free sugars is associated with an increased risk of obesity, dyslipidemia, and chronic metabolic diseases [15]. Furthermore, excessive consumption of total fat, saturated fat, and trans fat elevates blood cholesterol levels and cardiovascular risk [16]. These foods are typically energy-dense and nutrient-poor [17], leading to excessive caloric consumption and subsequent weight gain [18,19]. Several physiological mechanisms may underlie the link between ultra-processed food consumption and obesity, particularly in men. Ultra-processed foods, typically high in energy density, added sugars, and unhealthy fats, can disrupt appetite regulation and promote visceral fat accumulation [20], to which men may be more prone due to gender-specific differences in fat metabolism and hormonal responses [21,22]. This trend is especially pronounced in modern societies, where fast-paced lifestyles and the need for convenient meal solutions drive a greater reliance on such products.

As of 2023, the prevalence of obesity among Korean adults aged ≥19 years was 37.2%, showing an upward trend from 30.9% in 2014. The prevalence was notably higher in men (45.6%) than in women (27.9%) [23]. According to the World Health Organization (WHO), approximately 879 million adults worldwide were classified as obese by 2022, indicating that one in eight adults are affected by obesity. Compared to 1990, the global number of individuals with obesity has more than doubled, making obesity a critical and escalating public health issue associated with an increased risk of various chronic diseases and mortality. Obesity is strongly linked to cardiovascular diseases [24,25], type 2 diabetes [26], respiratory conditions [27], musculoskeletal disorders [28,29], and several types of cancer [30]. Additionally, it has significant implications for mental health, contributing to depression, low self-esteem, disordered eating, stress, and an overall reduction in quality of life [31,32]. Given that obesity is a complex, chronic condition requiring management from early life through adulthood, effective prevention and control strategies must involve not only individual-level interventions but also coordinated efforts from governments, communities, and international organizations.

With the expansion of the processed food market and rising consumption, there has been growing interest in the health and nutritional implications of ultra-processed foods as well as their association with various diseases. However, in Korea, existing research on ultra-processed food consumption has predominantly focused on adolescents and adults [33,34], with limited studies specifically targeting the adult population. Therefore, the objective of this study is to investigate the consumption patterns of ultra-processed foods among Korean adults aged 19–64 years, with a particular focus on gender-specific differences. Additionally, the study examines the associations between ultra-processed food intake, nutrient intake, and obesity. The findings aim to inform gender-sensitive dietary strategies and public health policies targeting nutritional health and obesity prevention.

## 2. Materials and Methods

### 2.1. Study Design and Participants

This study used data from the 8th (2019–2021) Korea National Health and Nutrition Examination Survey (KNHANES), a nationwide survey conducted in Korea. It is conducted annually in accordance with the National Health Promotion Act, surveying approximately 10,000 Koreans to assess their health status, health-related awareness and behaviors, and dietary intake. It provides nationally representative statistics and is used not only to evaluate the health status of the population but also to develop or improve health policies. The results of the KNHANES are also utilized in various research projects for international comparisons of health levels among countries and for studies on health promotion and disease prevention, as required by organizations such as the WHO and the Organization for Economic Co-operation and Development (OECD). In the second year of the 8th KNHANES, 2020, survey activities were partially suspended due to the outbreak of coronavirus disease 2019 (COVID-19). As a result, among the 192 sample enumeration districts nationwide, health interviews and examinations were conducted in 180 districts and nutrition surveys were completed in 166 districts, reflecting a slight decrease compared to the previous year [35].

For this study, data were derived from 22,559 participants of the 8th KNHANES, conducted between 2019 and 2021. Of these, 12,450 adults aged 19 to 64 years were selected for analysis after excluding 10,109 individuals who were either under 19 or over 65 years of age. Subsequently, 1488 participants with implausible total daily energy intake values (<500 kcal or >5000 kcal) were removed, resulting in a sample of 10,962 individuals. An additional 1300 participants were omitted due to missing data on covariates, including gender, household income, educational level, household types, smoking status, and alcohol consumption. Accordingly, the final analytic sample comprised 9662 individuals, consisting of 4105 men and 5557 women.

### 2.2. Study Variables

#### 2.2.1. Consumption of Ultra-Processed Foods

To analyze the status of ultra-processed food consumption by gender among the study participants, we classified the food codes collected from the 24 h recall data of the KNHANES’s nutrition survey according to the NOVA food classification system. According to the NOVA classification system, foods were categorized into NOVA 1 (unprocessed or minimally processed foods), NOVA 2 (processed culinary ingredients), NOVA 3 (processed foods), and NOVA 4 (ultra-processed foods). Foods classified as NOVA 4 include products that can be easily consumed as meal replacements, such as hamburgers, pizzas, cereals, and bread [36].

To analyze the relationship between nutrient intake and obesity among the study participants, the amount of ultra-processed food consumption was calculated, and tertiles of this amount were used in the analysis, classified into three groups according to the ultra-processed food intake.

#### 2.2.2. Dietary Assessment

To assess the dietary intake status according to ultra-processed food consumption by gender among the study participants, we utilized 24 h dietary recall data obtained using the structured dietary survey form employed in the KNHANES. In this method, trained dietitians conducted in-person interviews using the multiple-pass approach, which improves recall accuracy. Participants were asked to report all foods and beverages consumed during the previous 24 h, including details on food type, preparation method, brand name (where applicable), and portion size, with the help of a food portion booklet and standard measuring tools. The 24 h recall data reflect dietary intake on the day prior to the survey (from midnight to midnight of a given day). While this method captures short-term intake rather than usual long-term intake, it provides comprehensive and detailed information on the types and quantities of foods consumed, which is suitable for estimating the intake of ultra-processed foods in large population studies.

For macronutrients such as carbohydrates, proteins, and fats, the acceptable macronutrient distribution range (AMDR) was calculated to determine the appropriate intake levels. The acceptable range for adults aged 19 and older is 55–65% for carbohydrates, 7–20% for protein, and 15–30% for fat [32]. To understand the nutrient intake of the study participants, the daily intake of 14 nutrients, including carbohydrates, proteins, fats, dietary fiber, calcium, phosphorus, iron, sodium, potassium, vitamin A, vitamin B_1_, vitamin B_2_, niacin, and vitamin C, was calculated.

Additionally, to assess the consumption of ultra-processed foods, we referred to a previous study [5] and categorized the intake into 12 subgroups: (1) cereals, breads, cakes, sandwiches, etc., (2) distilled alcoholic beverages, (3) sugar-sweetened beverages, (4) fish and meat processed foods, (5) instant noodles and dumplings, (6) traditional sauce, (7) sweetened milk and its products, (8) others (instant sauce, condiments, etc.), (9) cookies, chips, and snacks, (10) soft drinks, fruit and vegetable drinks, (11) instant cooked rice, soup, and other dishes, and (12) confectionary.

#### 2.2.3. General Characteristics and Obesity Variables

To identify the demographic characteristics of the study participants, we examined their gender, average age, income level, marital status, household type, education level, smoking status, and alcohol consumption. Gender was categorized as ‘men’ and ‘women,’ and the average age was calculated for each group. Income level was classified according to the income level classification criteria of the KNHANES as ‘low’, ‘middle-low’, ‘middle-high’, and ‘high’. Household types were categorized as ‘single-person households’ or ‘multi-person households’ with two or more members. Education level was divided into ‘elementary school graduate or less’, ‘middle school graduate’, ‘high school graduate’, and ‘college graduate or higher’. Smoking status was classified as ‘past/non-smoker’ and ‘current smoker’, while alcohol consumption was defined as ‘no’ as lifetime non-drinker or less than one drink per month in the past year, and ‘yes’ as drinking more than one drink per month in the past year. Body mass index (BMI) was calculated as weight (kg) divided by height squared (m^2^).

In this study, the criteria for obesity were based on the definitions provided in KNHANES. Anthropometric measurements, including height and weight, were obtained by trained health professionals, following a standardized protocol. Obesity was defined as BMI ≥ 25 kg/m^2^.

### 2.3. Data Analysis

All data analyses were performed using SAS version 9.4 (SAS Institute, Cary, NC, USA), with statistical significance defined as *p* < 0.05. The sample from the KNHANES was designed under a complex sampling design, and analyses were conducted using the SURVEY procedure, which allows for the consideration of weights, strata variables (kstrata), and clustering variables (psu). To analyze differences in dietary intake according to gender-based ultra-processed food consumption, multiple linear regression analysis was conducted for continuous variables and chi-square tests were applied for categorical variables. All analyses were adjusted for gender, age, and educational level. Additionally, obesity prevalence according to gender-based ultra-processed food consumption was analyzed using multiple logistic regression models. Model 1 was unadjusted, Model 2 was adjusted for gender, age, and energy intake, and Model 3 included variables from Model 2 plus adjustments for income level, education level, smoking status, alcohol consumption, physical activity and energy intake. Adjusted odds ratios (AORs) and 95% confidence intervals (CIs) were calculated.

### 2.4. Ethical Considerations

The study protocol was approved by the Institutional Review Board of the Korea Disease Control and Prevention Agency (approval numbers: 2018-01-03-C-A, 2018-01-03-2C-A, 2018-01-03-5C-A).

## 3. Results

### 3.1. General Characteristics by Gender

Table 1 shows the general characteristics of the study participants according to gender. Among the 9662 participants, there were 4105 men (50.2%) and 5557 women (49.8%). Significant differences were found between the gender in terms of mean age, age distribution, marital status, household type, education level, smoking status, alcohol consumption, BMI, and overall obesity (all *p* < 0.01). The mean age was higher in women (42.6 years) than in men (41.9 years). In terms of age distribution, 22% and 21.5% of men were in their 20s and 30s, respectively, whereas for women, these percentages were 21.1% and 19.9%, respectively. The ratio equalized in the 40s (23.8% for both gender), and in the 50s and 60s, women (24.8% and 10.4%, respectively) had higher percentages than men (24.4% and 8.2%, respectively). Marital status showed that a higher percentage of women (74.8%) were married than men (64.2%). Single-person households were more common among men (12.3%) than women (6.9%). Regarding education level, 52.5% of the men and 50.5% of the women had at least a college degree. Current smokers and alcohol consumers were more prevalent among men (34.2% and 70.5%, respectively) compared to women (6.2% and 48.3%, respectively). BMI was higher in men (24.9 kg/m^2^) than in women (23.1 kg/m^2^), and men had higher rates of abdominal obesity (38.7%) and overall obesity (45.8%) compared to women (23.3%, 25.9%).

### 3.2. Status of Ultra-Processed Food Consumption by Gender

**Figure 1:**
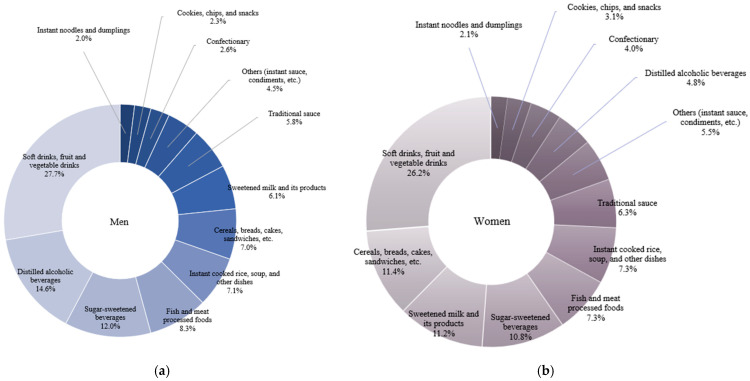
Relative contribution (%) of each ultra-processed food subgroup of study participants by gender in Korean adult. (**a**) Men. (**b**) Women. The 12 subgroups are (1) cereals, breads, cakes, sandwiches, etc., (2) distilled alcoholic beverages, (3) sugar-sweetened beverages, (4) fish and meat processed foods, (5) instant noodles and dumplings, (6) traditional sauce, (7) sweetened milk and its products, (8) others (instant sauce, condiments, etc.), (9) cookies, chips, and snacks, (10) soft drinks, fruit and vegetable drinks, (11) instant cooked rice, soup, and other dishes, and (12) confectionary.

Table 2 presents the consumption of ultra-processed foods and the 12 subcategories by gender, and Figure 1 shows the relative contribution. The study found that men consumed more ultra-processed foods than women (*p* < 0.0001). Significant differences between gender were observed in nine items: ‘Distilled alcoholic beverages,’ ‘sugar-sweetened beverages,’ ‘fish and meat processed foods,’ ‘instant noodles and dumplings,’ ‘traditional sauce,’ ‘sweetened milk and its products,’ ‘others (instant sauce, condiments, etc.),’ ‘soft drinks, fruit and vegetable drinks,’ and ‘instant cooked rice, soup, and other dishes’ (all *p* < 0.05). Both men and women consumed ‘soft drinks, fruit and vegetable drinks’ the most among the ultra-processed foods. Men consumed ‘distilled alcoholic beverages,’ ‘sugar-sweetened beverages,’ and ‘fish and meat processed foods’ in that order, while adult women consumed ‘cereals, breads, cakes, sandwiches, etc.,’ ‘sweetened milk and its products,’ and ‘sugar-sweetened beverages’ in that order.

### 3.3. Nutrient Intake According to Ultra-Processed Food Consumption by Gender

**Figure 2:**
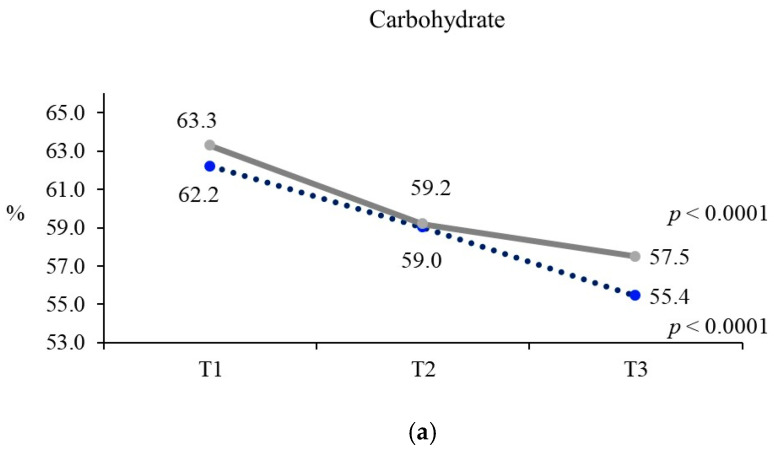

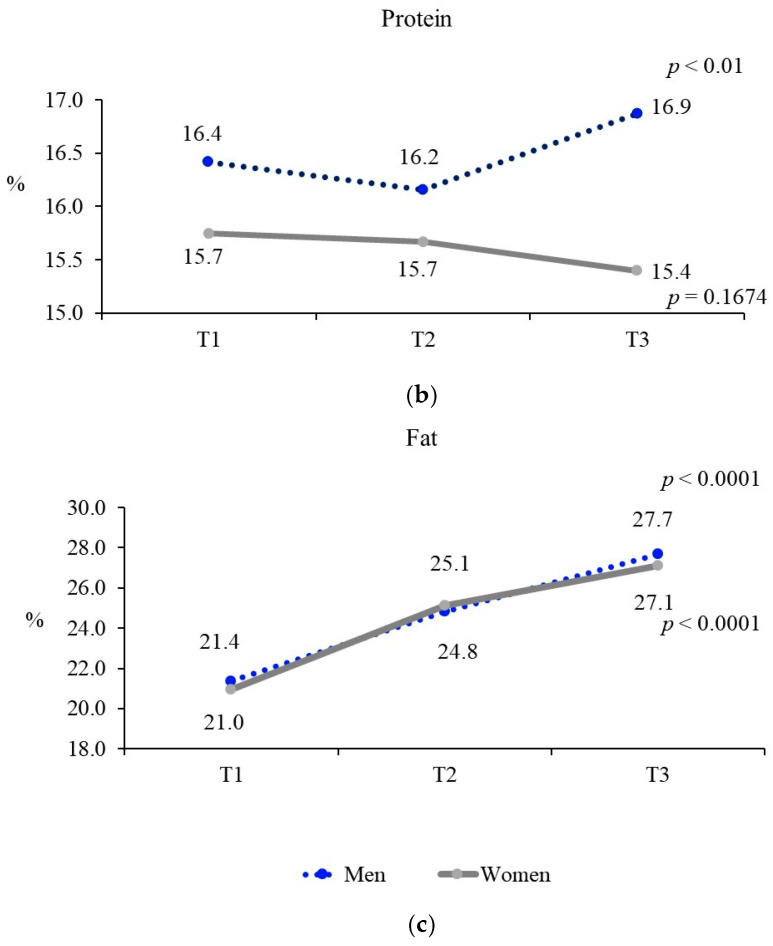
Acceptable macronutrient distribution range of study population according to the third tertile of ultra-processed food consumption by gender in Korean adults. Study participants were divided into tertiles based on their ultra-processed food intake (T1, T2, and T3). (**a**) Carbohydrate. (**b**) Protein. (**c**) Fat.

Figure 2 shows the AMDR by ultra-processed food consumption status, and Table 3 presents nutrient intake. Among men, there were significant differences in the proportions of carbohydrates, protein, and fat to total energy according to the level of ultra-processed food consumption (all *p* < 0.01). The group with the highest UPF intake had a lower proportion of carbohydrates but higher proportions of protein and fat than the lowest tertile group. For women, significant differences were observed in the proportions of carbohydrates and fat (*p* < 0.0001), with the highest tertile group showing a lower carbohydrate proportion and a higher fat proportion than the lowest tertile group. Nutrient intake analysis showed a trend in which higher ultra-processed food consumption correlated with increased nutrient intake in both men and women, showing significant differences in nutrient intake according to the level of ultra-processed food consumption (all *p* < 0.01).

### 3.4. Obesity Prevalence According to Ultra-Processed Food Consumption by Gender

Table 4 presents the prevalence of obesity according to ultra-processed food consumption status and gender. Model 1 was unadjusted, Model 2 was adjusted for gender, age, and energy intake, and Model 3 included Model 2 plus adjustments for education level, income level, marital status, household type, smoking status, alcohol consumption, and physical activity. In Model 3, among men, the group with the highest UPF intake had a 1.28-times-higher risk of obesity (AOR: 1.28; 95% CI: 1.05–1.55) than the lowest tertile group (*p* for trend = 0.0003).

## 4. Discussion

This study utilized data from the Korea National Health and Nutrition Examination Survey for three years (2019–2021) to examine the consumption patterns of ultra-processed foods by gender among adults aged 19–64 and to analyze the association between dietary intake and obesity.

A total of 9662 Korean adults were included in the final analysis, comprising 50.2% men and 49.8% women. Compared with men, women had a higher mean age, a greater proportion of married individuals, a higher rate of single-person households, and higher educational attainment. In contrast, current smoking and alcohol consumption rates were significantly higher in men. Heavy smokers tend to weigh more than nonsmokers or light smokers, likely due to lifestyle factors such as physical inactivity and unhealthy diets [37]. Smoking is known to reduce health-related quality of life [38] and increase central fat accumulation and insulin resistance and is a risk factor for metabolic syndrome, diabetes, and cardiovascular disease [39]. Alcohol consumption contributes to weight gain and obesity, leading to various adverse health outcomes [40,41]. These findings suggest that men are more vulnerable to obesity-related risk factors than women are.

There were notable gender-based differences in ultra-processed food consumption patterns. Men consumed more than 1.5 times the amount of ultra-processed food than women. Among the ultra-processed food subgroups, soft drinks and fruit and vegetable drinks were the most consumed by both gender, with the intake of men exceeding that of women by more than 1.5 times. Beverages are the leading contributors to added sugar intake among ultra-processed food [42], particularly carbonated drinks, fruit/vegetable juices, and coffee drinks [43]. These sugar-sweetened beverages contain high levels of artificial sweeteners and fructose, which can lead to rapid increases in blood glucose and insulin levels, followed by hypoglycemia and fat accumulation, thereby contributing to obesity [44,45]. In addition, many UPFs, particularly sweets and sugar-sweetened beverages, are likely to contain high-fructose corn syrup as a major source of added sugars [46,47]. Although the precise identification of high-fructose corn syrup content was limited by available food composition data, excessive fructose intake—especially from high-fructose corn syrup—is known to promote hepatic de novo lipogenesis, increasing the risk of non-alcoholic fatty liver disease [48,49]. This represents an additional metabolic pathway by which UPF consumption may adversely impact health beyond obesity. In men, a high intake of such beverages is a key risk factor for obesity [50], hypertension, diabetes, and cardiovascular diseases [51]. Gender-based consumption patterns [52] may also be explained by behavioral differences such as higher physical activity among men, leading to increased energy needs and greater beverage consumption [53,54]. Among the subgroups of ultra-processed foods, men consumed ‘distilled alcoholic beverages’ as the second most frequently consumed category, with intake levels more than four times higher than those of women. This markedly higher consumption among men suggests that they are particularly vulnerable to alcohol-related health risks. Distilled alcoholic beverages contain high concentrations of alcohol, and excessive intake is directly associated with a range of adverse health outcomes including liver disease, hypertension, cardiovascular disorders, and mental health problems [55,56,57]. For women, the second most consumed ultra-processed food subgroup was “cereals, breads, cakes, sandwiches, etc.,” followed by “sweetened milk and its products,” “sugar-sweetened beverages,” and “fish and meat processed foods,” highlighting a clear difference from the consumption patterns of men.

In addition to health considerations, the affordability, wide variety, convenience, and strong flavor profiles of UPFs have been shown to significantly influence food purchasing decisions across populations [58,59]. The wide availability and aggressive marketing of these products, particularly in modern retail environments, contribute to their growing consumption, especially among individuals with limited time and financial resources [59]. Such market dynamics represent a major challenge for public health interventions aimed at reducing UPF intake. These gender-based differences underscore the need for targeted policy intervention. For men, strategies should focus on raising awareness about the health risks associated with sugar-sweetened beverages and processed meats, aiming to reduce the high consumption of ultra-processed beverages. For women, campaigns that promote healthier choices of cereals and dairy products may be more effective. Therefore, gender-specific dietary improvement programs and health education are essential to promote nutritional balance and reduce ultra-processed food consumption.

Both men and women with high ultra-processed food consumption showed higher overall nutrient intake levels; however, this supports previous findings that a higher dietary energy contribution from ultra-processed food is associated with poorer nutrient intakes [34] and low dietary quality [59,60,61]. In addition, AMDR analysis showed that higher ultra-processed food consumption was associated with a lower proportion of carbohydrates, with men having a lower carbohydrate ratio than women. Among men, the highest tertile of UPF intake group had the highest protein proportion, which may be attributed to the relatively high intake of “fish and meat processed foods,” ranked fourth among the twelve ultra-processed food subgroups consumed by men. Furthermore, as ultra-processed food consumption increased, the proportion of fat increased in both gender, with men showing a higher fat ratio than women, potentially contributing to the higher prevalence of obesity in men.

Among men, the prevalence of obesity was 1.28 times higher (OR: 1.28) in the highest tertile of UPF intake group than in the lowest tertile group after adjusting for relevant variables. The association between ultra-processed food consumption and obesity has been well-documented in previous studies [19,62]; instant or ultra-processed foods are often characterized by high caloric density and poor nutritional quality, with essential nutrients frequently lacking. Moreover, physiological factors may partly explain the stronger association observed between ultra-processed food consumption and obesity in men compared to women. Gender hormones, particularly testosterone and estrogen, influence energy metabolism, fat distribution, and appetite regulation [63]. Men generally exhibit a greater visceral fat accumulation pattern and a higher propensity for central adiposity, which is more sensitive to excess caloric intake from ultra-processed foods [64]. In addition, some evidence suggests that men may experience greater hedonic responses and reward-driven eating behaviors toward ultra-processed foods, further exacerbating their risk of obesity [65]. The WHO classifies obesity as a form of malnutrition. While malnutrition is typically perceived to result from insufficient food intake, nutrient imbalance, such as excess calories with inadequate essential nutrients, can also lead to obesity. This interpretation highlights the fact that both underweight and obesity may reflect nutritional deficiencies. The rising prevalence of obesity ultimately imposes a socioeconomic burden, placing strain on the healthcare systems worldwide. Therefore, along with caloric restriction for obesity management, it is essential to implement nutrition education that ensures the adequate intake of essential nutrients.

Beyond obesity, emerging evidence indicates that high consumption of UPFs is associated with an elevated risk of developing other chronic diseases, including type 2 diabetes, cardiovascular disease, certain cancers, and depression [66,67]. This underscores the importance of reducing UPF intake not only as a strategy for obesity prevention but also as a critical component of broader efforts to combat noncommunicable diseases and promote long-term health in adult populations. The limitation of this study is its cross-sectional design, which precludes the establishment of causal relationships between UPF consumption and obesity prevalence among Korean adults. While we observed significant associations, the temporal sequence between exposure (UPF consumption) and outcome (obesity) cannot be determined. Therefore, the findings should be interpreted with caution, and future longitudinal or interventional studies are needed to confirm these relationships. In addition, the dietary data was self-reported, which may have led to recall bias. Despite applying a systematic approach to classify UPFs, the dietary assessment tool used in KNHANES was not specifically designed to measure UPF intake. Consequently, some degree of misclassification may have occurred. Nevertheless, this study is meaningful in that it highlights gender-based differences in UPF consumption among Korean adults, demonstrating that men consumed higher levels of UPFs than women and that obesity prevalence was significantly higher among men with greater UPF intake. Based on the findings of this study, it is important to develop dietary guidelines that promote the replacement of ultra-processed foods with unprocessed or minimally processed alternatives rather than simply aiming to reduce ultra-processed food consumption. Furthermore, given the association between ultra-processed food intake and obesity, particularly the gender-based differences observed, there is a need to develop tailored nutritional interventions and public health policies that reflect these gender-specific patterns.

## 5. Conclusions

This study utilized nationally representative data from the KNHANES from 2019 to 2021 to examine ultra-processed food consumption patterns by gender among Korean adults aged 19–64 years and analyze their association with dietary intake and obesity, a major chronic disease. The findings revealed clear gender differences in ultra-processed food consumption: Korean men consumed significantly more ultra-processed food than did women. Among the 12 subcategories of ultra-processed food, both men and women most frequently consumed ‘soft drinks, fruit and vegetable drinks’. Men also consumed high amounts of ‘distilled alcoholic beverages’, ‘sugar-sweetened beverages’, and ‘fish and meat processed foods’, whereas women consumed more ‘cereals, breads, cakes, sandwiches, etc.’, ‘sweetened milk and its products’, and ‘sugar-sweetened beverages’. Among men, those with the highest tertiles of UPF intake had a lower proportion of energy from carbohydrates and higher proportions of protein and fat than those with the lowest intake. Furthermore, the association between ultra-processed food consumption and obesity showed a significant trend in men, with higher ultra-processed food intake linked to an increased prevalence of obesity. These results highlight the need to improve the overall dietary patterns by encouraging the substitution of ultra-processed foods with unprocessed or minimally processed alternatives. Importantly, this study underscores the necessity of developing gender-specific strategies for obesity prevention tailored to the distinct dietary behaviors of men and women.

## Figures and Tables

**Table 1:** Sociodemographic characteristics of study participants by gender in Korean adults.

	Men (n = 4105)	Women (n = 5557)	Total (n = 9662)	*p*
Gender				
N (%)	4105 (50.2)	5557 (49.8)	9662 (100.0)	0.7384
Age (years)				
19–64 years	41.9 ± 0.2	42.6 ± 0.2	42.5 ± 0.3	0.0055
19–29	769 (22.0)	858 (21.1)	1627 (21.6)	0.0022
30–39	763 (21.5)	1020 (19.9)	1783 (20.7)	
40–49	972 (23.8)	1375 (23.8)	2347 (23.8)	
50–59	1011 (24.4)	1501 (24.8)	2512 (24.6)	
60–64	590 (8.2)	803 (10.4)	1393 (9.3)	
Income level				
Low	1039 (24.3)	1341 (23.0)	2380 (23.6)	0.3575
Middle-low	1018 (25.2)	1371 (24.9)	2389 (25.1)	
Middle-high	1025 (25.0)	1407 (25.8)	2432 (25.4)	
High	1023 (25.5)	1438 (26.3)	2461 (25.9)	
Marital status				
Married	2830 (64.2)	4459 (74.8)	7289 (69.5)	<0.0001
Single	1275 (35.8)	1098 (25.2)	2373 (30.5)	
Household types				
Single-person household	546 (12.3)	452 (6.9)	998 (9.6)	<0.0001
Multi-person household	3559 (87.7)	5105 (93.1)	8664 (90.4)	
Educational level				
Elementary school or less	146 (2.3)	343 (4.5)	489 (3.4)	<0.0001
Middle school	248 (4.6)	428 (6.1)	676 (5.4)	
High school	1620 (40.4)	2119 (38.9)	3739 (39.6)	
College or higher	2091 (52.7)	2667 (50.5)	4758 (51.6)	
Smoking status				
Paster/no smoker	2682 (65.8)	5245 (93.8)	7927 (79.8)	<0.0001
Current Smoker	1423 (34.2)	312 (6.2)	1735 (20.2)	
Alcohol consumption ^(1)^				
yes	2894 (70.5)	2574 (48.3)	5468 (59.4)	<0.0001
no	1211 (29.5)	2983 (51.7)	4194 (40.6)	
BMI (kg/m^2^)	24.9 ± 0.1	23.1 ± 0.1	23.3 ± 0.0	<0.0001
Obesity	1859 (45.8)	1504 (25.9)	3363 (35.9)	<0.0001

Values are presented as n (weighted %) or mean ± SE. All analyses were adjusted for gender, age, and educational level. ^(1)^ Alcohol consumption was defined with ‘no’ as lifetime non-drinker or less than one drink per month in the past year, and ‘yes’ as drinking more than one drink per month in the past year.

**Table 2:** Ultra-processed food and subgroup intake of study population by gender in Korean adults.

	Men (n = 4105)	Women (n = 5557)	*p*
Ultra-processed food intake	401.3 ± 8.0	260.1 ± 4.6	<0.0001
Ultra-processed food subgroup intake			
(1) Cereals, breads, cakes, sandwiches, etc.	28.1 ± 1.3	29.6 ± 1.2	0.3553
(2) Distilled alcoholic beverages	58.6 ± 2.9	12.4 ± 1.3	<0.0001
(3) Sugar-sweetened beverages ^(1)^	48.2 ± 2.3	28.1 ± 1.4	<0.0001
(4) Fish and meat processed foods	33.4 ± 1.7	19.0 ± 0.9	<0.0001
(5) Instant noodles and dumplings	8.0 ± 0.7	5.7 ± 0.4	0.0032
(6) Traditional sauce	23.4 ± 0.5	16.3 ± 0.3	<0.0001
(7) Sweetened milk and its products	24.6 ± 1.5	29.2 ± 1.5	0.0335
(8) Others (instant sauce, condiments, etc.)	17.5 ± 0.6	14.2 ± 0.5	<0.0001
(9) Cookies, chips, and snacks	9.2 ± 0.5	8.2 ± 0.4	0.0932
(10) Soft drinks, fruit and vegetable drinks	111.1 ± 4.5	68.1 ± 2.8	<0.0001
(11) Instant cooked rice, soup, and other dishes	28.4 ± 1.8	18.9 ± 1.1	<0.0001
(12) Confectionary	10.6 ± 0.7	10.5 ± 0.6	0.8433

Values are presented as mean ± SE. ^(1)^ Includes coffee or tea products with added sugar or milk, cocoa, or other sugar-sweetened beverages.

**Table 3:** Nutrient intakes of study population according to the tertile of ultra-processed food intake by gender in Korean adults.

	Men (n = 4105)				Women (n = 5557)			
	T1	T2	T3	*p*	T1	T2	T3	*p*
Energy (kcal)	1837.6 ± 20.7	2197.2 ± 21.7	2703.2 ± 25.0	<0.0001	1348.9 ± 12.6	1630.6 ± 15.0	1950.3 ± 18.4	<0.0001
Carbohydrate (g)	276.1 ± 3.1	305.8 ± 3.4	329.7 ± 3.6	<0.0001	209.7 ± 2.2	237.3 ± 2.4	265.3 ± 2.6	<0.0001
Protein (g)	74.0 ± 1.1	84.5 ± 1.1	101.0 ± 1.3	<0.0001	52.2 ± 0.7	62.8 ± 0.8	72.0 ± 1.0	<0.0001
Fat (g)	43.8 ± 0.9	59.4 ± 1.0	76.0 ± 1.3	<0.0001	31.6 ± 0.5	45.4 ± 0.7	58.2 ± 0.9	<0.0001
Dietary fiber (g)	26.3 ± 0.4	27.9 ± 0.4	28.2 ± 0.4	0.0024	21.8 ± 0.3	23.4 ± 0.3	23.4 ± 0.3	0.0001
Calcium (mg)	473.2 ± 8.3	546.7 ± 8.5	583.0 ± 9.5	<0.0001	378.0 ± 5.8	472.3 ± 7.0	526.3 ± 7.8	<0.0001
Phosphorus (mg)	1086.9 ± 13.9	1221.1 ± 13.6	1356.7 ± 15.2	<0.0001	820.0 ± 9.8	959.5 ± 10.7	1054.7 ± 11.9	<0.0001
Iron (mg)	9.6 ± 0.2	11.1 ± 0.2	13.0 ± 0.3	<0.0001	7.2 ± 0.1	8.8 ± 0.1	10.1 ± 0.2	<0.0001
Sodium (mg)	3501.3 ± 56.5	3916.9 ± 58.0	4360.3 ± 64.3	<0.0001	2333.1 ± 34.4	2885.9 ± 41.2	3141.2 ± 43.2	<0.0001
Potassium (mg)	2800.1 ± 38.4	3049.8 ± 37.6	3168.3 ± 39.1	<0.0001	2283.3 ± 29.7	2562.9 ± 32.7	2626.0 ± 33.1	<0.0001
Vitamin A(μg RAE)	380.5 ± 13.5	432.3 ± 10.9	469.8 ± 15.7	<0.0001	324.9 ± 6.7	406.6 ± 11.7	422.8 ± 8.9	<0.0001
Vitamin B_1_ (mg)	1.2 ± 0.0	1.3 ± 0.0	1.6 ± 0.0	<0.0001	0.9 ± 0.0	1.0 ± 0.0	1.1 ± 0.0	<0.0001
Vitamin B_2_ (mg)	1.6 ± 0.0	1.9 ± 0.0	2.2 ± 0.0	<0.0001	1.2 ± 0.0	1.5 ± 0.0	1.7 ± 0.0	<0.0001
Niacin (mg)	12.9 ± 0.2	14.8 ± 0.2	18.1 ± 0.3	<0.0001	9.3 ± 0.1	11.1 ± 0.2	12.8 ± 0.2	<0.0001
Vitamin C (mg)	54.5 ± 1.6	65.3 ± 2.2	88.1 ± 6.5	<0.0001	57.7 ± 1.6	65.6 ± 2.2	71.8 ± 2.2	<0.0001

Values are presented as mean ± SE. Study participants were divided into tertiles based on their ultra-processed food intake (T1, T2, and T3).

**Table 4:** Association between ultra-processed food intake and obesity of study population according to the tertile of ultra-processed food consumption by gender in Korean adults.

	Men (n = 4105)				Women (n = 5557)			
	T1	T2	T3	*p* for Trend	T1	T2	T3	*p* for Trend
Obesity								
Unadjusted	1.00	1.02 (0.86–1.21)	1.16 (0.98–1.38)	<0.0001	1.00	0.87 (0.73–1.04)	0.77 (0.64–0.92)	<0.0001
Model 2	1.00	1.05 (0.88–1.25)	1.26 (1.03–1.54)	<0.0001	1.00	0.95 (0.79–1.15)	0.98 (0.81–1.18)	<0.0001
Model 3	1.00	1.03 (0.88–1.24)	1.28 (1.05–1.55)	0.0003	1.00	1.02 (0.83–1.23)	1.02 (0.84–1.24)	<0.0001

Values are presented as adjusted odds ratio (95% confidence interval). Study participants were divided into tertiles based on their ultra-processed food intake (T1, T2, and T3). Multiple logistic regression analysis was performed to estimate the odds for the tertiles of ultra-processed food intake for the study participants by gender. Model 2 was adjusted for gender, age and energy intake. Model 3 was adjusted for gender, age, education level, income level, marital status, household type, smoking status, alcohol consumption, physical activity and energy intake.

## Data Availability

The data that support the findings of this study are openly available in the Korea National Health and Examination Survey at http://knhanes.cdc.go.kr, accessed on 2 April 2025.
